# Modulation of DNA Damage and Repair Pathways by Human Tumour Viruses

**DOI:** 10.3390/v7052542

**Published:** 2015-05-22

**Authors:** Robert Hollingworth, Roger J Grand

**Affiliations:** School of Cancer Sciences, College of Medicine and Dentistry, University of Birmingham, Birmingham B15 2TT, UK; E-Mail: rxh291@student.bham.ac.uk

**Keywords:** DNA damage, DNA repair, human tumour viruses, HPV, MCPyV, HTLV-1, EBV, KSHV, HBV, HCV

## Abstract

With between 10% and 15% of human cancers attributable to viral infection, there is great interest, from both a scientific and clinical viewpoint, as to how these pathogens modulate host cell functions. Seven human tumour viruses have been identified as being involved in the development of specific malignancies. It has long been known that the introduction of chromosomal aberrations is a common feature of viral infections. Intensive research over the past two decades has subsequently revealed that viruses specifically interact with cellular mechanisms responsible for the recognition and repair of DNA lesions, collectively known as the DNA damage response (DDR). These interactions can involve activation and deactivation of individual DDR pathways as well as the recruitment of specific proteins to sites of viral replication. Since the DDR has evolved to protect the genome from the accumulation of deleterious mutations, deregulation is inevitably associated with an increased risk of tumour formation. This review summarises the current literature regarding the complex relationship between known human tumour viruses and the DDR and aims to shed light on how these interactions can contribute to genomic instability and ultimately the development of human cancers.

## 1. Introduction

Over the past two decades interest in the relationship between viruses and the DNA damage response (DDR) has increased exponentially. For all the commonly studied viruses, at least some investigations have been undertaken into the effect of viral infection, or expression of individual viral genes, on activation of the host DDR. Similarly, the ways in which viruses impinge on pathways responsible for DNA repair have been the subject of multiple investigations. While most attention has focused on DNA viruses [1,2,3], there is also a growing body of literature concerning both RNA viruses and retroviruses, whose lifecycle includes a DNA intermediate [4,5,6].

Historically, interest in the relationship between viruses and DNA damage originates from observations made half a century ago, that infection with a number of virus species could lead to extensive chromosomal damage [7]. It was subsequently found that, in the case of adenovirus, herpes simplex virus 1 (HSV-1), and human cytomegalovirus (HCMV), chromosomal breaks induced by viruses frequently occur at specific sites [8]. More recently, as knowledge of the DDR has developed, increasing layers of complexity concerning viral/DDR interactions have been revealed. It is now clear that some aspects of the DDR can represent a potent antiviral defence that may be disabled following entry to the host cell. In other cases, viruses take advantage of DDR activation to modulate the cell cycle while specific proteins are hijacked to aid viral replication. 

Over the past few years a number of excellent reviews of the virus/DDR relationship have been published [1,9,10,11]. Some of these have focused on particular aspects of the DDR whereas others have concentrated on particular viral species. Here we have tried to draw together the relevant published literature specific to viruses associated with human tumours *in vivo*, including relevant RNA viruses as well as those with a DNA genome. Thus we have summarised the literature concerning human papilloma virus (HPV), Merkel cell polyomavirus (MCPyV), human T-cell leukemia virus type 1 (HTLV-1), Epstein-Barr virus (EBV), Kaposi's sarcoma-associated herpesvirus (KSHV), Hepatitis B virus (HBV) and Hepatitis C virus (HCV) (Table 1). By adopting this approach it is hoped that an appreciation can be gained of how the interaction between a virus and the DDR may contribute to its oncogenic potential and, ultimately, to cancer development.

## 2. Human Tumour Viruses

Human oncogenic viruses have been defined as necessary but not sufficient to initiate cancer. It is now apparent that only in relatively rare cases do oncogenic viruses in isolation give rise to tumours in otherwise healthy individuals. For example, while the majority of the population carries latent persistent EBV infection, EBV-derived tumours are comparatively rare. In the case of KSHV, although most infected individuals remain asymptomatic, when the immune system is incapacitated by HIV, this herpesvirus can induce Kaposi’s sarcoma in a significant proportion of patients. In the case of other viruses, which are present in a latent state, it seems that multiple genetic events, often attributable to environmental factors, are required to initiate tumour development. As outlined here, it is probable that genomic instability induced during the viral lifecycle plays a significant role in tumour initiation. Regardless of the mechanisms of tumourigenesis, it is estimated that between 10% and 15%, of human malignancies are attributable to viral infection [12] and viruses are now second only to smoking as a leading cause of theoretically preventable cancer. This review will focus on genetic instability introduced by infection with oncogenic viruses, and how these pathogens subsequently modulate pathways responsible for recognition and repair of DNA damage.

## 3. The DNA Damage Response (DDR)

The DDR is the response of the cell to lesions in its genome that occur during normal cellular processes or are introduced by exogenous agents. The inability to repair this damage can result in a variety of acquired clinical conditions such as cancer and neurodegeneration [13,14]. In addition, several inherited disorders are associated with defects in genes crucial for proper functioning of the DDR. Here we include a brief outline of a very complex and fast developing area of biological research; mention is made of only a limited number of proteins involved in the DDR which are relevant to the subsequent discussion. 

### 3.1. Sensors and Transducers

The cellular response to DNA damage is primarily regulated by the activities of three phosphatidylinositol 3-kinase-like kinases (PIKKs): ataxia telangiectasia mutated (ATM), ATM and Rad3-related (ATR) and DNA-dependent protein kinase (DNA-PK) [15,16,17]. ATM and DNA-PK are principally activated in response to double strand breaks (DSBs) while ATR can be activated following stalled replication forks and by DNA lesions that result in persistent single stranded DNA (ssDNA). Subsequent phosphorylation of transducer and effector molecules by these kinases results in cell cycle arrest, DNA repair and, potentially, apoptosis or senescence. 

**Figure 1:**
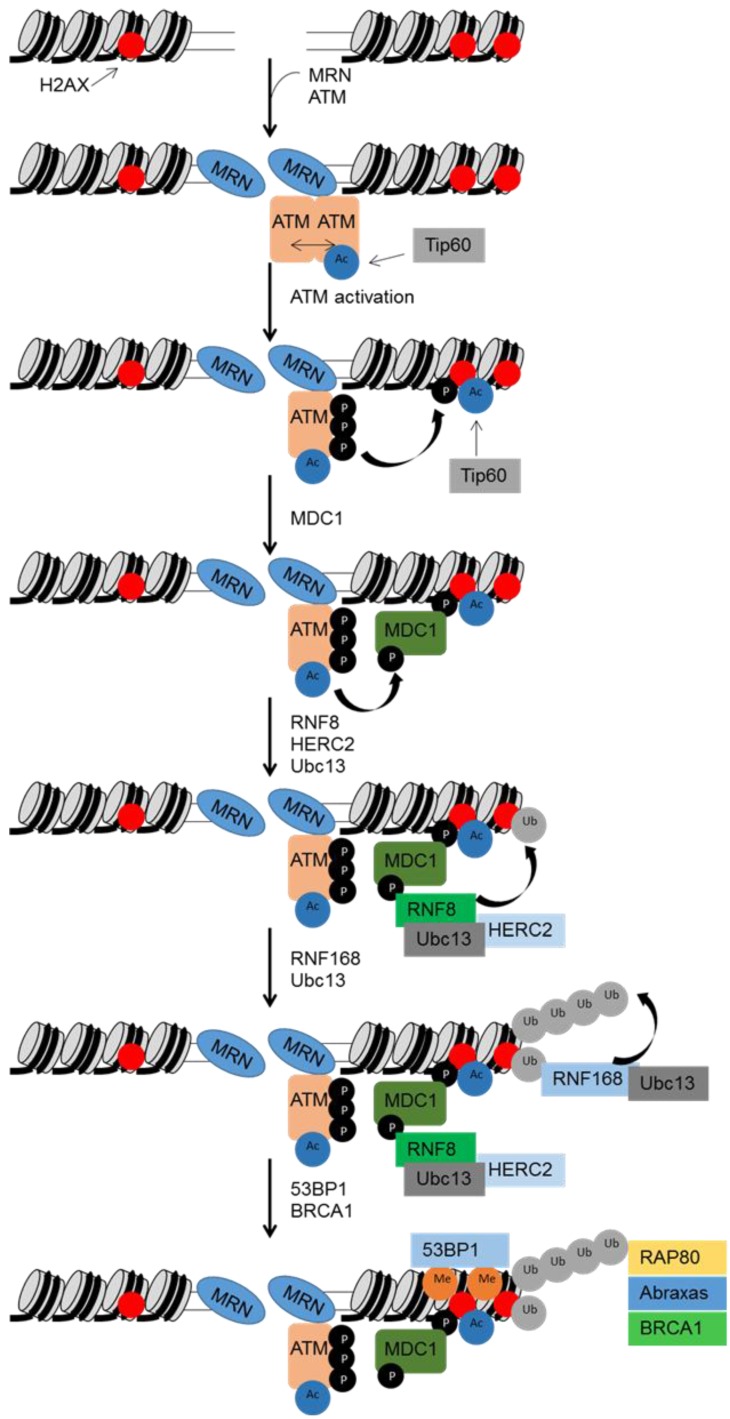
ATM activation in response to DSBs. The MRN complex rapidly migrates to sites of DSBs and, along with the acetyltransferase Tip60, contributes to activation of ATM kinase activity. Phosphorylation of histone H2AX by ATM results in binding of MDC1 which subsequently mediates recruitment of factors, such as 53BP1 and BRCA1, which participate in DSB repair and regulation of cell cycle checkpoints.

DSBs are recognised by the MRN complex (comprising Mre11, Rad50 and NBS1), which mediates the recruitment and activation of ATM [18,19,20] (Figure 1). The activation of ATM also requires acetylation by TIP60, and autophosphorylation that results in disassociation of the inactive dimer to an active monomer [21,22]. ATM can phosphorylate a large number of downstream targets that include CHK2 and H2AX. The phosphorylation of histone H2AX (giving rise to γH2AX) at sites of DSBs allows binding of MDC1, which acts as a scaffold for the formation of large protein complexes spreading appreciable distances from the site of the actual break [23]. MDC1 promotes recruitment of ubiquitin ligases, such as RNF8 and RNF168, which in turn facilitate binding of 53BP1 and BRCA1 [24,25,26]. DSBs also lead to activation of DNA-PK, which is involved in the non-homologous end joining (NHEJ) repair pathway summarised below.

**Figure 2:**
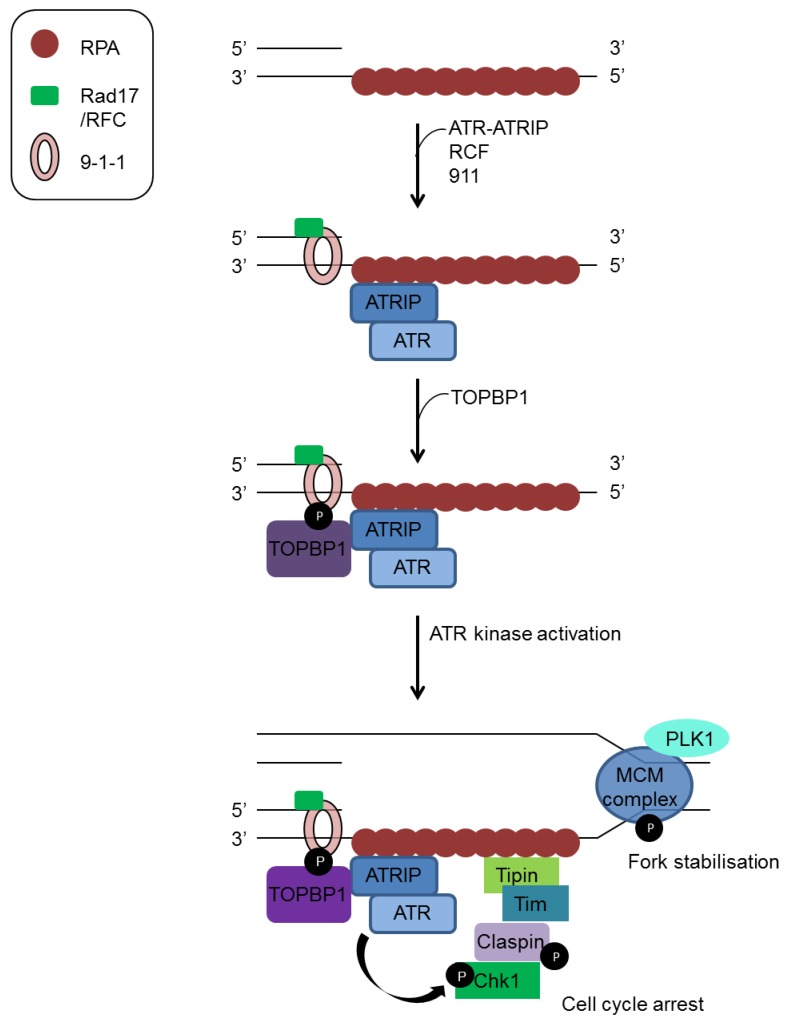
ATR activation in response to stalled replication forks. ATR and ATRIP bind to stretches of ssDNA coated with RPA while the Rad17-RFC complex independently loads the 9-1-1 checkpoint clamp onto ssDNA/dsDNA junctions. Subsequent recruitment of TOPBP1 mediates activation of ATR while Claspin and the Timeless (Tim)/Tipin complex facilitate CHK1 activation.

The stalling of replication forks and resection of DSBs can generate stretches of ssDNA that provide a signal for activation of the ATR kinase (Figure 2) [17]. The ssDNA is bound by RPA which recruits ATR via its binding partner ATRIP. The generation of ds/ssDNA junctions can promote loading of the Rad9-Rad1-Hus1 (9-1-1) complex by the Rad17-RFC complex [27]. The recruitment of TOPBP1 then facilitates ATR activation which subsequently phosphorylates CHK1 with the help of the adaptor protein Claspin [28]. The Timeless-Tipin complex also contributes to CHK1 activation by interacting with both RPA and Claspin [29]. Activation of the ATR-CHK1 pathway is able to slow replication by inhibition of replication origin firing and initiate cell cycle check-points by phosphorylation of the Cdc25 phosphatases [17,30].

### 3.2. Activation of Cell Cycle Checkpoints

**Figure 3:**
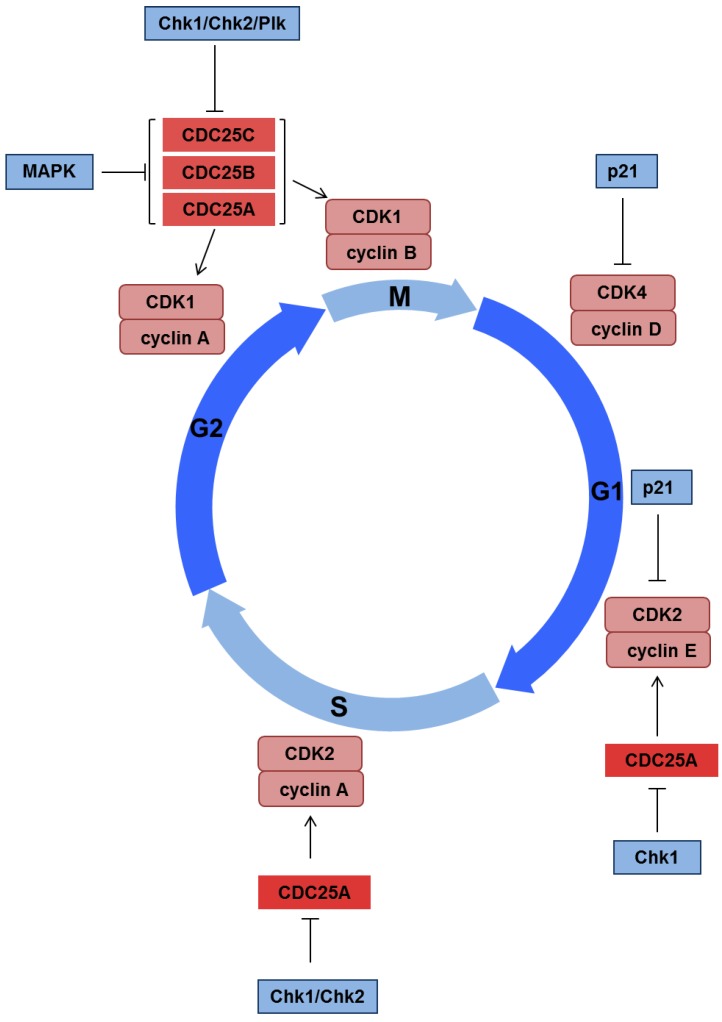
Regulation of the cell cycle by cyclins and CDKs. In response to DNA damage phosphorylation of CHK1 and CHK2 can lead to degradation of Cdc25 phosphatases. Cdc25 degradation inhibits activation of CDKs and delays progression of the cell cycle. Increased expression of p21 by p53 following DNA damage can also mediate cell cycle arrest through inhibition of CDKs.

A typical consequence of DDR activation is the triggering of cell cycle arrest that prevents replication of potentially mutagenic lesions and provides opportunity for DNA repair [31]. Three major checkpoints are involved in monitoring the integrity of the genome under normal circumstances. The G1/S checkpoint is activated to prevent the initiation of cellular replication in the presence of DNA damage. The intra-S-phase checkpoint can slow or arrest DNA synthesis following replication stress or DNA damage that occurs during DNA replication. Finally, the G2/M checkpoint is activated to ensure that damage incurred during S and G2 is not segregated during mitosis (Figure 3). 

Activation of ATM and ATR leads to phosphorylation of CHK1 and CHK2, which in turn regulate downstream targets, such as p53, Cdc25 and Wee1. The phosphorylation of the Cdc25 phosphatases leads to their degradation, which prevents the activation of cyclin-dependent kinases (CDKs) causing cell cycle delay. The key transcriptional target following p53 stabilization is p21, which can impede G1/S transition through inhibition of the cyclin E/Cdk2 complex. The retinoblastoma protein (Rb) also has a key role in cell cycle progression by binding the E2F transcription factors and inhibiting progression into S-phase. In addition, activation of the tyrosine kinase Wee1 following DNA damage leads to inhibition of the cyclin B/Cdk1 complex preventing G2/M transition.

Many viruses have evolved mechanisms of disrupting the G1/S checkpoint to allow S-phase progression, which is often required for replication of their genetic material. The best characterised of these is disassociation of the Rb/E2F complex by the small DNA tumour viruses (adenovirus, HPV, simian virus 40 [SV40]) allowing E2F-mediated transcription of viral genes [32]. Several viruses, such as human immunodeficiency virus (HIV), can also activate the G2/M checkpoint to prevent mitotic entry or to maintain a pseudo S-phase state [33]. This can be achieved via several mechanisms including prevention of nuclear entry of the cyclin B/Cdk1 complex, activation of the ATR-CHK1 pathway or manipulation of Wee1 and Cdc25C activities [33].

### 3.3. Apoptosis and Senescence

While cell cycle arrest and DNA repair are typical consequences of DDR activation, failure to properly correct DNA lesions can lead to cellular senescence or apoptosis. The activation of apoptotic pathways following DNA damage typically occurs via p53 and defects in this response are associated with tumorigenesis. ATM, ATR, CHK1, and CHK2 can all directly phosphorylate p53 preventing its degradation and increasing its transcriptional activity [34]. While p53 can play a protective role by arresting the cell cycle and upregulating DNA repair pathways, it can also induce transcription of pro-apoptotic genes, such as PUMA, NOXA and BAX [35]. Since apoptosis of the host cell can reduce the opportunity for viral propagation, many viral species have evolved mechanisms to inhibit programmed cell death and promote cellular survival [36]. For example, oncogenic herpesviruses encode homologs of BCL-2, an anti-apoptotic protein that prevents the release of cytochrome c from mitochondria [37,38]. HPV E6 meanwhile promotes the ubiquitination and degradation of p53, as well as other pro-apoptotic proteins, such as BAK, FADD and c-Myc [39,40]. Lymphocytes expressing the HTLV-1 Tax protein have elevated levels of c-FLIP, a protein that can inhibit apoptosis mediated by the CD95 death receptor [41]. 

Persistent activation of the DDR can also lead to cellular senescence whereby the cell remains viable but replicative capacity is lost. A DDR that results in cellular senescence can be triggered by the progressive shortening of telomeres, repetitive DNA sequences that protect chromosome ends, following successive cell divisions [42]. As this provides an important safeguard against malignant proliferation, the interference of tumour viruses with cellular mechanisms involved in the maintenance of telomeres could play a role virally-induced oncogenesis. To promote elongation of telomeres, and thus replicative immortality, viruses can both activate telomerase enzymes and stimulate a more error-prone alteRNAtive lengthening of telomere (ALT) pathway [43]. Cellular proliferation induced by viruses can lead to progressive shortening of telomeres and this has been observed in HPV-associated cervical carcinoma and in hepatocarcinomas containing HBV and HCV [44,45]. To counteract this effect, HPV and HBV oncoproteins have been shown to increase expression and activity of hTERT, the catalytic subunit of telomerase [46,47]. There is also evidence of activation of the ALT pathway in human embryonic fibroblasts immortalized by HPV E6 and E7 and also in endothelial cells immortalised by KSHV vGPCR [48,49]. It is also conceivable that DNA damage induced by viruses could cause irreparable damage to telomeric DNA that, if the senescence response is compromised due to deregulation of cell cycle checkpoints, may lead to telomere dysfunction and genomic instability [43]. 

**Table 1:** Known human tumour viruses. *Includes well-characterised proteins rather than an exhaustive list.

Virus	Genome	Viral Oncoproteins *	Associated Cancer
Human papilloma virus (HPV)	dsDNA	E6 and E7	Cervical cancer, penile cancer, anogenital carcinoma, head and neck cancer
Merkel cell polyoma virus (MCPyV)	dsDNA	Large T antigen	Merkel cell carcinoma (MCC)
Human T cell leukaemia virus-1 (HTLV-1)	ssRNA	Tax	Adult T cell leukaemia (ATL)
Epstein Barr virus (EBV)	dsDNA	EBNA2, EBNA3C, LMP-1, LMP-2	Nasopharyngeal carcinoma (NPC), Burkitt’s lymphoma, Hodgkin’s lymphoma, post-transplant lymphoproliferative disease (PTLD), T cell lymphoma, gastric cancer
Kaposi’s sarcoma-associated herpesvirus (KSHV)	dsDNA	LANA, v-cyclin, vGPCR, vIL-6, vBcl-2, vFLIP, Kaposin B	Kaposi’s sarcoma (KS), primary effusion lymphoma (PEL), multicentric Castleman’s disease (MCD)
Hepatitis B virus (HBV)	ssDNA + ssRNA	HBx	Hepatocellular carcinoma (HCC)
Hepatitis C virus (HCV)	ssRNA	Core, NS3, NS5A	Hepatocellular carcinoma (HCC), B-cell lymphoma

## 4. DNA Damage Repair Pathways

As DNA lesions can take many forms, multiple DNA repair pathways have evolved to correct specific type of DNA damage. A brief overview of the principle mechanisms of DNA repair pathways are included below. In addition, the interactions between human tumour viruses and key components of these repair pathways are summarised in Table 2.

**Table 2:** Viral interactions with components of core DNA repair pathways. This list is not exhaustive but includes observations mentioned in this article. VRCs—Viral replication centres.

Repair Pathway	DDR Target Protein	Virus/Viral Protein	REFERENCE
Direct repair	MGMT	HPV E6	[50]
Base excision repair (BER)	XRCC1 Β polymerase	HPV E6 HTLV-1 Tax	[51] [52]
Nucleotide excision repair (GG-NER)	XPC PCNA DDB1 TFIIH subunits (XPB, XPD)	MCPy LT HTLV-1 Tax EBV BPLF1 HBx HBx	[53] [54] [55] [56,57] [58,59]
Mismatch repair (MMR)	MSH2, MSH6 MSH2, MSH6, MLH1, hPSM2	KSHV VRCs HTLV-1 Tax EBV VRCs	[60] [61] [62]
Single strand break repair	PARP-1 XRCC1	KSHV VRCs HPV E6	[60] [51]
Homologous recombination (HR)	Rad51 BRCA1 Rad52	HPV16 E7 EBV VRCs HPV31 VRCs HPV31 VRCs HTLV-1 VRCs EBV VRCs	[63] [64] [65] [65] [66] [64]
Non-homologous end joining (NHEJ)	DNA-PKcs Ku70/Ku80	KSHV VRCs HTLV-1 VRCs KSHVorf59 KSHV VRCs	[60] [66] [67] [60]

### 4.1. Non-Homologues End Joining (NHEJ)

**Figure 4:**
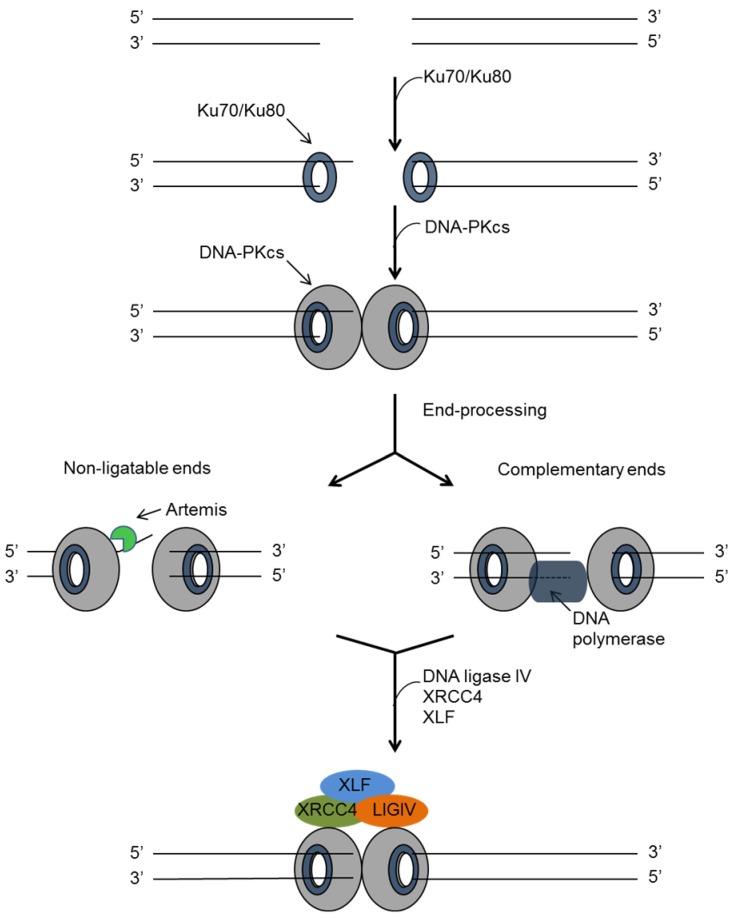
Non-homologous end joining repair of DSBs. Ku70/80 binds to DNA ends and provides a scaffold for the recruitment of additional proteins such as DNA-PKcs. If required, DNA ends may be cleaved by the Artemis nuclease and gaps filled by DNA polymerases. Finally, the ligation complex consisting of XLF, XRCC4 and DNA Ligase IV ligates the DNA ends to complete the repair.

Double-strand breaks in DNA can be repaired by non-homologous end joining (NHEJ) that operates throughout the cell cycle (Figure 4). DNA ends are first recognised and bound by the Ku70/80 heterodimer which recruits the catalytic subunit of DNA-PK (DNA-PKcs) [68,69]. Subsequent phosphorylation by DNA-PK results in its disassociation from the site of damage. End processing involves Artemis, an endonuclease, together with polynucleotide kinase/phosphatase (PNKP), DNA polymerase µ (Pol µ) and DNA polymerase λ (Pol λ). These are required to synthesise complementary nucleotides to fill in single strand overhangs [70]. DNA-PK also facilitates recruitment of the NHEJ ligation complex, comprising DNA Ligase IV, XRCC4 and XLF, which is required for the final joining of the DNA strands [71,72]. 

### 4.2. Homologous Recombination (HR)

**Figure 5:**
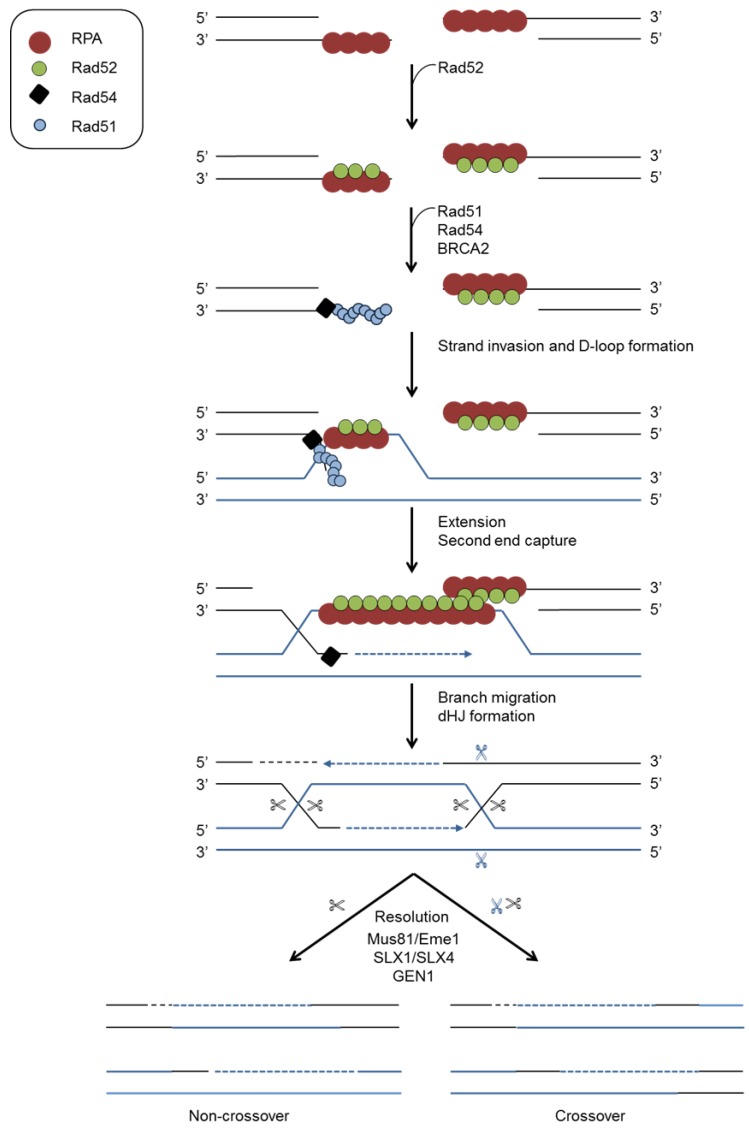
Homologous recombination repair of DBSs. DNA end resection results in ssDNA that is first coated by RPA. Rad51, in conjunction with Rad52 and BRCA2, then displaces RPA. Rad51 and Rad54 catalyse strand invasion and homology search with the undamaged template. Following DNA synthesis via polymerases, the resulting Holliday junctions are resolved.

Repair of double strand breaks by homologous recombination (HR), which occurs in the S and G2 phases of the cell cycle, is considered more accurate than NHEJ because an undamaged sister chromatid is used as a template (Figure 5). During HR, DNA ends are processed to form 3′ ssDNA tails that are bound by RPA. This requires the endonuclease activity of the MRN component Mre11 together with CtIP [73,74]. Additional resection involves the combined activities of exonuclease-1 (EXO1), DNA replication helicase 2 (DNA2) and Bloom Syndrome protein (BLM) [24]. The recombinase Rad51, together with BRCA2 and Rad52, displaces RPA and regions of homology on the sister chromatid are located through the action of Rad52 [70,75,76]. A Rad51 nucleoprotein complex with Rad54 locates homologous regions in the undamaged dsDNA template. Strand invasion which leads to the formation of a displacement loop is followed by DNA synthesis by DNA polymerases such as Pol δ using the homologous DNA as a template. HR can be completed either by the synthesis-dependent strand annealing pathway (SDSA) or the DSBR pathway through a process of “second end capture” facilitated by Rad52 [77]. During SDSA the newly synthesised strand is displaced by RTEL helicase, annealing with single ssDNA on the other side of the break. Second end capture gives rise to double Holliday junctions which are resolved to separate sister chromatids. This resolution requires the endonucleases Mus1/Eme1 and SLX1/SLX4 and the resolvase GEN1 [78].

### 4.3. Single Strand Break Repair

Single strand breaks (SSBs) are much more common than DSBs although they are often converted to DSBs which are more deleterious to the cell [79]. SSBs can arise from a variety of sources such as the action of reactive oxygen species (ROS) and the erroneous incorporation of ribonucleotides into DNA. They can also arise indirectly during the process of excision repair and as a result of the activity of DNA topoisomerase 1. SSBs are generally recognised by poly (ADP-ribose) polymerase 1 (PARP1), which is activated by DNA strand breaks and then ribosylates both itself and a number of target proteins [80]. Poly (ADP-ribose) chains are rapidly degraded by poly (ADP-ribose) glycohydrolase (PARG) after SSB repair is completed. PARP1 is responsible for recruitment of XRCC1, to the site of damage. This acts as a scaffold for the accumulation of further factors such as polynucleotide kinase/phosphatase (PNKP), aprataxin, and Polβ. Depending on how the SSB has arisen, and therefore the structure of the 3′ and 5′ DNA ends, different enzymes are involved in processing and repairing the break. For example, PNKP and AP endonuclease 1 (APE1) modify 3′ ends whereas 5′ termini are substrates for DNA polymerase β [81]. After processing of the damaged termini, gaps in the DNA are filled by PoIβ, PoIδ and PoIε. However, other proteins, such as XRCC1, PARP1, FEN-1 and proliferating cell nuclear antigen (PCNA) may also contribute to this process [79]. As a final step, the DNA is ligated by Ligase 3α (short patch repair) or Ligase 1 (long patch repair) [82,83,84]. 

### 4.4. Base Excision Repair (BER)

Lesions and mutations, which affect single bases, are removed by the base excision repair pathway (BER). Adducts in DNA tend to occur following exposure to alkylating agents and ROS. Certain modifications, such as O^6^-methyl guanine, 1-methyl adenine and 3-methyl cytosine can be repaired directly by alkyltransferases and DNA dioxygenases [85,86]. During BER, damaged DNA bases resulting from single base loss or base oxidation are recognised and removed by one of a number of DNA glycosylases which cleave the bond linking the base to the sugar-phosphate backbone [87]. Removal of the base and end processing by the AP endonuclease 1 (APE1) results in formation of an apyrimidinic/apurinic (AP) site and a SSB. The single nucleotide gap is filled by Polβ and the DNA religated by Ligase 3 and XRCC1 [88]. AlteRNAtive BER pathway components, such as PNKP, may come into play depending on the specific DNA glycosylases involved [89].

### 4.5. Nucleotide Excision Repair (NER)

Nucleotide excision repair (NER) is used to repair DNA which contains large helix-distorting adducts such as cyclo-butane pyrimidine dimers (CPD) and 6, 4 pyrimidine-pyrimidone photoproducts formed as a result of UV irradiation [90]. Two forms of NER are recognised: global genome NER (GG-NER), which is used to generally repair lesions, and transcription-coupled NER (TC-NER), which is used for repair of transcriptionally active DNA [91]. During GG-NER, lesions are recognised by the Xeroderma-pigmentosum C (XPC)-RAD23B-centrin 2 complex. Recruitment of TFIIH, containing the helicases XPB and XPD and the endonucleases XPG and ERCC1-XPF results in opening of the DNA double helix and cleavage and removal of the aberrant base. The gap is filled by Polδ and Polɛ in the presence of PCNA and the DNA is religated by Ligase 1 or Ligase 3 [92]. During TC-NER, the lesion is detected by RNA polymerase II (Pol II) when it becomes stalled during elongation [93]. This results in the recruitment of a large protein complex which includes Cockayne Syndrome proteins, ERCC6 and ERCC8, certain core NER factors as well as TC-NER specific proteins UVSSA, USP7 and HMG14 [91]. Repair then proceeds by a similar mechanism to that of GG-NER.

### 4.6. Mismatch Repair (MMR)

The mismatch repair (MMR) pathways are activated to deal with DNA base mispairings which arise during DNA replication, generally due to errors of DNA polymerases [94]. Two MMR complexes detect mismatched DNA: MutSα, comprising MSH2 and MSH6, recognises mismatched insertions/deletions 1–2 bases long and MutSβ, comprising MSH2 and MSH3, recognises longer insertion/deletion mispairs [95]. The MutLα-ATPase complex (MLH1 and PMS2) is recruited to the MutSα complex on the mismatched DNA with PCNA and RPA [96]. PMS2 is an endonuclease which, together with EXO1, is required for excision of the mismatched region [97]. The gap is filled by DNA Pol δ using the sister strand as a template and the DNA religated by Ligase 1, together with PCNA [96]. Whilst this simplified account explains MMR *in vitro*, it appears that *in vivo* other chromatin remodelling/modification factors and epigenetic modifications of histones are involved [98].

### 4.7. Fanconi Anaemia (FA) Pathway

The Fanconi anaemia (FA) pathway comprises between 15 and 20 proteins and is activated in response to interstrand cross-links (ICLs) [99,100]. ICLs arise following exposure to chemicals such as cisplatin and mitomycin C and result in the covalent cross linking of two DNA strands, inhibiting transcription and replication. The ICL is recognised by a FA anchor complex containing a number of proteins such as FANCM [99]. Subsequent recruitment of the FA core complex, comprising eight proteins, leads to monoubiquitination of FANCD2 and FANCI. This monoubiquitination results in the recruitment and activation of nucleases, such as FANCP and FANCQ, which cleave the DNA and “unhook” the cross-link. HR proteins are engaged in the later stages of ICL repair to resolve DSBs which are generated [99]. 

### 4.8. DNA Repair Pathways and the Cell Cycle

Although the DNA repair pathways outlined above have evolved to deal with specific types of DNA damage, their activity can vary significantly during different phases of the cell cycle [101]. As detailed above, DSB repair by HR is restricted to S and G2 phases due to the requirement of a template sister chromatid that allows faithful repair of the damaged region. The other primary DSB repair pathway, NHEJ, can occur throughout the cell cycle but is more prevalent in G1 due to the unavailability of HR. The CtIP protein has been identified as an important factor in stimulating HR since its phosphorylation by CDKs in S and G2 phases promotes its role in the initial resection step of HR [102]. Proteasomal degradation of CtIP in G1 may also contribute to inhibiting HR during this phase [103]. 

While the principle excision repair pathways can operate throughout the cell cycle, activity can also vary in different phases. For example, MMR is more prevalent during S-phase to correct replication errors while NER plays a key role in G1 to remove bulky lesions that could block DNA polymerases [101,104]. It has also been shown that the activities of key BER enzymes are higher in G1 following IR-induced DNA damage compared with the G2 phase [105]. S-phase is also associated with DNA damage tolerance pathways (DDT) that allow replication to proceed in the presence of unrepaired DNA damage. Lesions can be bypassed in an error-prone manner using specialised translesion synthesis (TLS) DNA polymerases, or more accurately by template switching (TS) which employs the sister chromatid as a template [106]. Since viruses can specifically interact with proteins involved in both DNA repair and cell cycle regulation, it is worthwhile to consider possible cell cycle effects when evaluating the efficiency of DNA repair pathways during viral infection.

The following text summarises the published literature concerning how the pathways detailed above are activated or subverted by viruses known to cause tumours in humans (Table 2). 

## 5. Human Papillomaviruses (HPV)

Human papillomaviruses (HPV) are small double stranded DNA viruses of approximately 8 kb that target the mucosal and cutaneous epithelium. HPV infection is associated with malignancies of the anogenital tract and the oropharynx and is a particular risk factor for the development of cervical cancer [107]. Over 100 HPV strains have been identified although only a limited number have been classified as high-risk based on their potential to cause disease. Among these high risk types, type 16, 18, 31, and 33 are responsible for approximately 90% of all cervical cancers [108]. 

HPV initially establishes infection in undifferentiated and actively proliferating cells in the basal layer of epithelium where progression of the viral lifecycle is tightly linked to cellular differentiation. In undifferentiated cells, the viral genomes are maintained as extra-chromosomal nuclear episomes, which are replicated in synchrony with cellular DNA. Upon cellular differentiation, the HPV genome is amplified to produce infectious particles and the cell cycle is deregulated to aid viral replication. In HPV-containing cancer cells, the viral episome is often lost and in high grade lesions HPV DNA is typically found integrated into the host genome [109]. 

The HPV genome can be divided into an early region that comprises six open reading frames (ORFs) designated E1 to E7, a noncoding region and a late region comprising two ORFs, L1 and L2, that transcribe major and minor capsid proteins respectively [110]. The E1 and E2 viral proteins are necessary for initiation of HPV replication while E6 and E7, highly expressed during genome amplification, are the primary oncoproteins required for malignant transformation. E6 and E7 are known to promote degradation of the tumour suppressors, p53 and retinoblastoma protein (pRB) respectively, while disruption of the E2F/RB complex by E7 drives progression of the cell cycle [111,112]. 

### 5.1. Activation of the DDR by HPV

Expression of the viral helicase E1 in several high-risk HPV types can induce a DDR that principally comprises activation of the ATM-CHK2 pathway [113,114,115,116]. Activation of ATM has been attributed to the induction of DSBs in cellular DNA caused by E1 which specifically requires the ATPase and dsDNA melting activity of the protein [116]. ATM activation by E1 has also been shown to induce cell cycle arrest in S and G2 phases leading to suppression of cell growth [114,116]. Nuclear export of E1 prevents DDR activation and cell cycle arrest in undifferentiated keratinocytes and the formation of a complex with E2 limits DDR activation while E1 is in the nucleus [114]. In several studies a subset of E1-expressing cells were found to contain phosphorylated CHK1 suggesting that HPV may induce cell cycle-dependent activation of the ATR pathway [113,115,117].

While a number of studies have focused on the ability of E1 to induce DNA damage directly, it has also been demonstrated that the coexistence of HPV episomes and integrated HPV DNA in some cells can lead to unscheduled DNA replication and host genomic alterations [117,118]. The re-replication of integrated HPV18 DNA can generate heterogeneous replication intermediates that recruit DNA repair proteins and activate the ATM-CHK2 pathway [117]. In this study, DDR activation was ascribed to the recognition of viral DNA structures rather than the direct induction of DNA damage by viral proteins, although E1 expression was still required to initiate HPV replication.

Expression of E7 has also been shown to cause DNA damage, which can subsequently activate the Fanconi anemia (FA) repair pathway in squamous cell carcinomas (SCCs) [119,120,121]. FANCD2 foci, a marker of FA activation, have been observed in SSC tissues while FANCD2, FANCD1 and BRCA2 are recruited to chromatin in HPV16 E7-expressing cells [120]. Consequently, expression of E7 in cells deficient in the FA pathway leads to unrepaired DNA damage and increases the risk of genetic instability. 

### 5.2. Involvement of DDR Factors in HPV Replication

Several studies have examined a role for DDR activation and individual DDR proteins in replication of HPV DNA [65,113,115]. It has been suggested that HPV-induced ATM activation and subsequent cell cycle arrest can provide a more suitable environment for viral replication in differentiating cells [113]. ATM inhibition during HPV31 infection was shown to adversely affect late viral genome amplification but not episomal maintenance [113]. In the same study it was demonstrated that CHK2 activity is required for capase activation, which subsequently plays a role in viral replication through cleavage of E1. 

Numerous DNA damage and repair proteins have been observed associated with HPV replication centres suggesting a role for these factors in the viral lifecycle. Levels of the DSB markers γH2AX and 53BP1 are elevated in HPV31-positive cells compared with uninfected cells and both can localize to HPV DNA foci along with phosphorylated ATM and CHK2 [65]. In addition, HR factors such as Rad51, BRCA1 and phosphorylated RPA have been seen associated with HPV31 replication centres suggesting that HR may be involved in HPV replication [65]. ATR pathway proteins ATRIP and TOPBP1 have also been observed at HPV18 replication sites [116,117]. TOPBP1 interacts directly with HPV E2 and suppression of this interaction results in an inability of the virus to establish episomes and reduces overall viral DNA replication [122]. 

### 5.3. Deregulation of DDR Signalling by E6 and E7

As well as activating the cellular DDR and recruiting DDR factors to replication centres, HPV also interferes with several DNA repair pathways, principally through the actions of E6 and E7. Expression of HPV16 E7 can increase persistence of γH2AX and Rad51 foci following IR-induced DNA damage in head and neck cancer cells [63]. While E7 expression can increase overall levels of the HR protein Rad51, the normal kinetics of DNA repair including the resolution of γH2AX foci are impeded. 

E6 from at least three HPV types interacts with XRCC1, a scaffold protein involved in BER, impairing the ability of E6-expressing cells to repair SSBs [51]. E6 also interacts with O^6^methylguanine-DNA methyl transferase (MGMT), a protein that protects against harmful mutations by repairing DNA adducts, and promotes its ubiquitin-dependent degradation [50]. Expression of HPV16 E6 was found to interfere with the recovery of fibroblasts from UV radiation by deregulating CHK1 activity [123] while HPV5 and 8 E6 proteins can promote degradation of the histone acetyl transferase p300. Degradation of p300 by E6 was shown to reduce ATR protein levels and subsequently increase thymine dimer persistence and DSB formation following UV exposure [124].

### 5.4. Summary

HPV activates the ATM pathway primarily through the expression of E1, although other viral proteins as well as viral DNA structures can also simulate DDR signalling. Several components of the ATM and ATR signalling pathways, as well as DNA repair factors, are localised to HPV DNA foci and may contribute to viral replication. Despite the presence of DNA damage, E6 and E7 promote cellular proliferation and survival by deregulating the cell cycle and impairing the apoptotic response. 

By driving cellular proliferation in the presence of unrepaired DNA damage and abrogating repair mechanisms, the introduction of genomic instability is an inevitable consequence of the HPV lifecycle that increases the chances of malignant transformation of the host cell. In addition, it has been demonstrated that the introduction of DSBs in cellular DNA leads to an increase in HPV16 integration events [125]. Since viral integration is frequently observed in HPV-related malignancies, it is conceivable that DNA damage induced by HPV proteins can facilitate viral integration which subsequently drives HPV-mediated carcinogenesis [126].

## 6. Merkel Cell Polyomavirus (MCPyV)

Merkel cell polyomavirus (MCPyV), the most recent human tumour virus to be identified, is responsible for approximately 80% of cases of the aggressive skin cancer known as Merkel cell carcinoma (MCC) [127]. As with other polyomarviruses, MCPyV has a small double-stranded DNA genome divided into early and late coding regions separated by a non-coding control region (NCCR). The early region produces a transcript whose alteRNAtive splicing results in the translation of three proteins; a large T antigen (LT), a small T antigen (sT) and the 57kT antigen. Three capsid proteins, VP1, VP2 and VP3, are encoded by the late region. In MMC tumours positive for MCPyV, the viral genome is integrated prior to clonal expansion of the cancer cells, strongly suggesting a viral driver of oncogenesis [128].

Similar to other polyomarviruses, MCPyV sT is involved in viral replication and targets the protein phosphatase 2A (PP2A). MCPyV sT may also play a role in regulation of the innate immune response and has been implicated in the development and progression of MCC [129,130]. Most attention, however, has focused on the multifunctional LT protein which contributes to initiating replication of the viral genome and also modulates a range of host cellular processes, some of which are analogous to the actions of SV40 LT [131]. In MCPyV-positive MCC, mutations in the carboxy terminus of LT result in production of a truncated LT protein that lacks the helicase activity required for viral replication but retains oncogenic properties, such as an ability to inactivate Rb [131,132]. Since exposure to UV is a known risk factor for the development of MCC and pyrimidine dimer substitutions are common among LT mutations, it is speculated that UV-induced DNA damage plays a role in carcinogenesis [131]. 

### 6.1. Activation of the DDR by MCPyV

It has been demonstrated that activation of both the ATM and ATR pathways occurs following MCPyV infection [133]. Activation of both pathways was observed following infection with native MCPyV virions and after viral genomes were directly introduced into cells via transfection. Expression of LT alone led to activation of ATR but not ATM while comet assays revealed that LT expression can cause DNA damage in the host cell. Use of various LT mutants indicated that the C-terminal region of the protein was responsible for both ATR activation and DNA damage. Activation of ATR by LT *C*-terminal region led to p53 phosphorylation, cell cycle arrest and suppression of cellular proliferation. The authors concluded that the C-terminal region of LT can act as a brake on cellular proliferation via DDR activation which offers a possible explanation as to why mutations in this region can contribute to development of MCC.

### 6.2. Involvement of DDR Factors in MCPyV Replication

It was recently shown that in MCPyV-infected cells, several DDR proteins associated with both the ATM and ATR pathways localise to foci containing MCPyV LT [134].Using immuno-FISH and BrdU staining to visualise viral DNA, it was found that DDR factors including ATR, phosphorylated CHK2, and γH2AX co-localised with LT at sites of active MCPyV replication. This localisation was abolished following introduction of a replication-defective virus or a virus containing a mutated origin of replication. Experiments using DDR kinase inhibitors and small interfering RNA (siRNA) suggested that the ATR kinase is important for efficient viral replication.

### 6.3. Interference with DNA Repair by LT

It has been demonstrated that MCC cells containing MCPyV have impaired ability to respond to UV-induced DNA damage compared to uninfected MCC cells [53]. These effects were attributed to a defect in the GG-NER pathway, in particular reduced expression of the XPC protein in the MCPyV-positive cells. Ectopic expression of both wild type and mutated LT inhibited G1 arrest following UV exposure but only expression of the mutated protein led to defects in DNA repair. The results suggest that mutated LT could increase genomic instability leading to MCC and provide a possible explanation for the higher incidence of MCC on sun-exposed skin.

### 6.4. Summary

Similar to SV40 LT [135], expression of MCPyV LT can activate a cellular DDR. But while SV40 LT interferes with downstream activation of p53, the DDR elicited by MCPyV LT leads to cell cycle arrest in a p53-dependent manner. In this way, checkpoint activation may provide selective pressure for generation of the truncated LT protein that fails to activate a growth-suppressive DDR. Truncated LT can also interfere with DNA repair of UV damage that could further contribute to genomic instability and tumorigenesis. During MCPyV infection, DDR factors are localised to sites of viral replication centres while ATR activation plays a positive role in viral replication. It has also been reported that ATM can directly phosphorylate LT although the functional significance of this has yet to be fully determined [136].

Investigations into the interaction between MCPyV and the DDR are clearly at an early stage although findings from closely related polyomarviruses, as well as other tumour viruses, have provided a basis for current research projects. Future studies may uncover how exactly the virus benefits from activation of the DDR and assess the relevant contribution of DDR dysregulation and other factors to the development of MCPyV-associated MCC. 

## 7. Epstein-Barr Virus (EBV)

Epstein–Barr virus (EBV) is a ubiquitous gammaherpesvirus carried by over 90% of the human population [137]. EBV has a dsDNA genome of approximately 170 kbp, primarily targets resting B lymphocytes and epithelial cells and is implicated in the development of human malignancies that include Burkitt’s lymphoma, Hodgkin’s lymphoma, nasopharyngeal carcinoma and gastric carcinoma [138]. 

The EBV lifecycle consists of two distinct states: latent infection and lytic replication. Following initial infection, the virus can establish life-long latency in the host where it is typically maintained as an extra-chromosomal episome that is replicated synchronously with cellular DNA by host polymerases. Latency is characterised by the expression of a tightly restricted subset of genes required for viral genome persistence. EBV latency transcripts include three membrane proteins (LMPs), six nuclear proteins (EBNAs), two non-coding RNAs (EBERs) and several microRNAs (miRNAs) [139]. The expression pattern of latent viral genes is variable among EBV-associated tumours and has led to the classification of four latency programmes known as Latency 0, I, II and III [139]. 

In a subset of cells, EBV can enter the lytic replication programme in which the viral genome can be amplified 100- to 1000-fold. The method of genome replication is distinct from latency in that there is greater requirement for replication proteins encoded by the virus. The immediate–early genes BZLF1 and BRLF1 are expressed first and encode transactivator proteins. These proteins then mediate expression of an ordered cascade of early and late viral genes that culminates in release of progeny virus from the host cell. Proteins expressed in both the latent and lytic stages have unique interactions with the host DDR.

### 7.1. Genetic Instability Induced by EBV Proteins

Several reports have demonstrated that EBV infection can lead to genetic instability in host cells through the introduction of DNA damage and dysregulation of DNA repair mechanisms. EBV-infected Burkitt’s lymphoma cell lines have elevated levels of γH2AX in the absence of exogenous damaging agents as well as an increased number of chromosomal abnormalities [140]. EBV nuclear antigen 1 (EBNA-1), expressed during latency and present in all EBV-associated malignancies, has been shown to induce chromosomal aberrations and DSBs through the production of ROS via NADPH oxidase activation [141,142]. Latent membrane protein 1 (LMP1), known to be essential for EBV-induced transformation of lymphocytes, can induce micronucleus formation and repress DNA repair in epithelial cells [143,144]. It was shown that LMP1 can stimulate the PI3K/Akt pathway resulting in phosphorylation of FOXO3a [144]. FOXO3a phosphorylation causes its retention in the nucleus impairing its ability to activate target genes such as DDB1, a component of the NER pathway.

Several EBV lytic proteins have also been shown to contribute to genetic instability in host cells. In nasopharyngeal carcinoma cells, EBV terminase BALF3 can induce DNA strand breaks and micronuclei formation and recurrent expression leads to tumorigenic features such as enhanced cell migration and invasiveness [145]. The EBV-encoded kinase BGLF4 is also associated with genetic instability [146]. BGLF4 expression leads to DDR activation that interferes with host DNA replication and delays the progression through S-phase [146]. Expression of the EBV DNase BGLF5 in epithelial cells can induce DSBs leading to an increase in microsatellite instability and genetic mutations [147]. BGLF5 expression also results in the down-regulation of several DNA repair genes involved in pathways such as MMR, NER, BER, and HR. Finally, BPLF1, a late tegument protein, deubiquitinates PCNA resulting in reduced recruitment of DNA polymerase η to sites of stalled replication where it would normally play a role in the translesion synthesis (TLS) DNA damage tolerance pathway [55]. 

### 7.2. Involvement of DDR Factors in EBV Replication

The role of the DDR proteins in EBV lytic replication has been studied by several research groups although the findings have not always been consistent. During lytic replication in B cells, EBV can elicit an ATM-mediated DDR with phosphorylation of H2AX, CHK2 and p53 observed following induction of the lytic cycle [148]. Phosphorylated ATM, NBS1 and Mre11 were found localised to sites of viral replication while downstream p53 signalling was inhibited. Inhibition of ATM activation by caffeine (a general PIKK inhibitor) during this investigation did not affect production of infectious virus. Two subsequent studies by the same group found that MMR proteins, such as MSH2, MSH6, MLH1, and hPSM2, as well as HR factors, including RPA, Rad51 and Rad52, are also localised to EBV replication compartments [62,64]. The HR factors, as well as PCNA and Mre11, were shown to be loaded onto newly synthesised viral DNA while knockdown of RPA and Rad51 significantly reduced viral replication. These findings point to a key role for individual DNA repair factors and the HR pathway in synthesis of EBV DNA. An interaction has also been reported between the early lytic protein BZLF1 and 53BP1, a protein that usually forms foci at the sites of DSBs [149]. Knockdown of 53BP1 inhibited production of progeny virus indicating that the protein is involved in EBV replication, possibly by protecting exposed DNA ends during genome amplification.

In a mechanism believed to be conserved among herpesviruses, the EBV lytic protein BGLF4 activates the acetyl transferase TIP60, a known regulator of the DDR through acetylation of ATM [150]. This interaction subsequently triggers an ATM-mediated DDR that plays a positive role in lytic replication of EBV. In contrast to previous reports, viral replication was suppressed in a dose-dependent manner following application of a specific ATM inhibitor. A further study provided evidence that ATM activation is required for efficient expression of EBV early lytic genes but not for viral DNA replication [151]. This study presented a model in which cellular stress activates the ATM pathway leading to activation of the EBV early lytic BZLF1 promoter by ATM substrates that can modify viral chromatin. More recently it has been shown that ATM activation during EBV lytic replication leads to phosphorylation of the transcription factor Sp1 [152]. Sp1 is subsequently localised to sites of replicating viral DNA along with other DDR factors associated with the ATM pathway. Phosphorylation of Sp1 was shown to mediate the recruitment of EBV replication proteins to viral DNA, highlighted by the fact that knockdown of Sp1 dramatically suppressed viral replication.

### 7.3. Dysregulation of Cell Cycle Checkpoints during EBV Infection

It has also been demonstrated that EBV-induced cellular proliferation can activate cell cycle checkpoints that lead to DDR signalling [153,154]. Activation of the ATM-CHK2 pathway has been observed following hyper-proliferation of primary B cells containing EBV [153]. In this context the DDR can act as an anti-tumour barrier as inhibition of ATM and CHK2 significantly increased the efficiency of B cell transformation by EBV. The study also demonstrated that expression of the EBV nuclear antigen 3C (EBNA3C) latent protein could attenuate DDR signalling that would otherwise arrest the growth of EBV-infected cells. 

EBNA3C can also interact directly with CHK2 and disrupt the G2/M cell cycle checkpoint induced by treatment with nocodazole which blocks cells in mitosis [155]. The binding of EBNA3C to CHK2 leads to phosphorylation of Cdc25c at Ser^216^. This causes Cdc25c to be localised to the cytoplasm allowing cyclin B/Cdc2 activation and progression through the G2/M checkpoint. EBNA3C has subsequently been shown to form a complex with H2AX facilitating its degradation via the ubiquitin proteasome pathway [156]. In lymphoblastoid cell lines (LCLs), H2AX knockdown resulted in down-regulation of the tumour suppressor p53 and increased expression of the oncoprotein Bub1. In addition to the actions of EBNA3C, expression of LMP1 in nasopharyngeal epithelial cells impairs the G2 checkpoint through defective CHK1 activation resulting in increased chromosomal instability [157].

Activation of the ATR pathway has also been demonstrated following oncogene-driven cellular replication induced by EBV infection [154]. EBV can interrupt downstream ATR signalling by increasing expression of STAT3, which leads to the loss of Claspin due to caspase 7 activity. Since Claspin is involved in mediating CHK1 phosphorylation by ATR, its loss leads to relaxation of the intra S-phase checkpoint, which would normally act as a barrier against EBV-driven cellular proliferation. 

### 7.4. Summary

Activation of the DDR has been observed in EBV-infected B cells and epithelial cells, during both the latent and lytic stages of the viral lifecycle. The cause of this DDR activation has been ascribed to several sources including the introduction of damage and inhibition of DNA repair, recognition of viral DNA as host cell damaged DNA, and aberrant cellular proliferation induced by viral proteins. In addition, the virus can interfere with cell cycle checkpoints to allow continued cellular proliferation in the presence of DNA damage. EBV also appears to recruit several DDR factors, including HR proteins, to replication sites where they may participate in viral DNA synthesis.

While several studies have noted the activation of ATM following lytic reactivation of EBV, there have been diverging opinions regarding the role of this kinase in viral replication. Contradictory observations could be the result of using different cell types, varying methods of inducing viral replication or the use of non-specific ATM inhibitors. While question marks remain over the role of the DDR during the lytic cycle, it has been shown that DDR factors contribute to viral genome replication during latency. For example, Mre11 and NBS1 are recruited to the origin of plasmid replication (OriP) in S-phase and play a role in maintenance of viral episomes through the formation of recombination junctions [158]. In summary, EBV appears to take advantage of DDR activation and utilise certain DDR proteins while disabling other aspects of the DDR that could lead to growth suppression and apoptosis. In view of the prevalence of EBV infection, the multiple interactions of the virus with the DDR and its ability to damage host cell DNA, it is perhaps surprising that EBV-associated tumours are not more common.

## 8. Kaposi’s Sarcoma Related Virus (KSHV)

Kaposi's sarcoma-associated herpesvirus (KSHV), also known as human herpesvirus 8 (HHV8), is the second gammaherpesvirus after EBV to be identified as a causative agent of human cancers. KSHV can infect and transform endothelial cells leading to the development of the angioproliferative malignancy known as Kaposi’s sarcoma [159]. In addition, KSHV is associated with B cell malignancies, primary effusion lymphoma (PEL), and multicentric Castleman's disease (MCD) [160,161]. Despite its association with cancer, KSHV infection is largely asymptomatic, with disease most likely to occur only following suppression of the host immune system. 

As with EBV, the KSHV lifecycle is biphasic and consists of latent and lytic stages. KSHV latency-associated nuclear antigen (LANA) is a multifunctional viral protein expressed in all latently infected cells and present in all KSHV-positive KS tumours [162]. LANA plays a vital role in tethering the KSHV episome to host DNA ensuring its distribution to daughter cells following cellular division [163]. LANA can also interact with multiple host cell proteins and has been implicated in tumorigenesis through interference with cellular pathways associated with cell cycle control, apoptosis, gene expression and immune regulation [164]. In contrast to the latent programme, KSHV lytic replication involves expression of a large set of viral genes necessary for production of new infectious progeny. Both latent and lytic genetic programmes have been implicated in the development of KSHV-related malignancies [165].

### 8.1. DDR Activation during Latent KSHV Infection

KSHV infection induces phosphorylation of H2AX and elevated levels of γH2AX have been observed in latently infected B cells [166,167]. LANA can interact with H2AX at its C terminus leading to formation of γH2AX, which is important for episomal persistence [166]. KSHV infection of primary endothelial cells was shown to result in phosphorylation of H2AX and ATM as early as 30 minutes after viral entry with γH2AX co-localising with viral genomes [167]. Transient phosphorylation of DDR effector kinases CHK1 and CHK2 was observed but downstream phosphorylation of Cdc25c was unaffected. Total H2AX levels also increased during KSHV infection, which was attributed to a decrease in K^48^-linked polyubiquitination and an increase in K^63^-linked polyubiquitination. Inhibition of ATM or depletion of H2AX has a negative effect on the ability of the virus to establish latency, suggesting that KSHV may mediate activation of the ATM arm of the DDR while inhibiting downstream signalling.

Ectopic expression of v-Cyclin, a homolog of mammalian D-type cyclins expressed during latency, leads to activation of the ATM pathway and S-phase arrest in endothelial cells [168]. This DDR acts as a block to cellular proliferation and could create selective pressure for the acquisition of mutations that abrogate this barrier while providing a growth advantage to cells defective in DDR components. Expression of v-Cyclin also leads to the introduction of chromosomal instability through disruption of the centrosome cycle resulting in multinucleation and aneuploidy [168,169,170]. Cells latently infected with KSHV can also bypass a G2/M checkpoint block induced by nocodazole [171]. It was shown that LANA can interact directly with CHK2 leading to activation of the cyclin B/Cdc2 complex that mediates progression through the G2/M checkpoint.

### 8.2. Involvement of DDR Factors in KSHV Replication

DNA affinity purification combined with mass spectrometry has been used to identify host cell factors that bind to KSHV origins of lytic replication (*ori-Lyt*) [60]. The proteins identified included the MSH2/MSH6 heterodimer involved in MMR and DNA-PKcs, PARP-1, and Ku80/70, all involved in NHEJ. Several of these proteins were also found to localise to viral replication compartments suggesting a role in viral replication. Treatment of KSHV-infected B cells with a PARP-1 inhibitor has been shown to enhance lytic replication but further analysis has also suggested that PARP-1 may play a positive role in replication of viral DNA, although it can negatively regulate late assembly of infectious virions [60,172].

### 8.3. DDR Activation during Lytic Replication of KSHV

Elevated levels of phosphorylated H2AX have been observed following initiation of KSHV lytic replication [67,173]. This observation has been attributed to the introduction of DSBs in cellular DNA by the viral mRNA export factor orf57 [173]. It was shown that sequestration of the human Transcription and Export complex (hTREX) by orf57 can cause newly transcribed mRNA to form R-loops through annealing to the DNA template strand which in turn leads to formation of DSBs. Another lytic protein, the processivity factor orf59, was shown to interact with Ku70 and Ku80 and impair NHEJ repair of DSBs [67]. Although KSHV lytic replication can activate the DDR, the lytic protein viral interferon regulatory factor 1 (vIRF1) has been shown to interact with ATM and impair DDR activation following etoposide treatment [174]. In addition, vIRF1 can interact with p53 and facilitate its proteasomal degradation. 

### 8.4. Summary

As with EBV, KSHV infection activates the ATM pathway and latently-infected cells display elevated markers of DNA damage and chromosomal aberrations. DDR activation appears to play a positive role in establishing and maintaining latency but does not result in cell cycle arrest or apoptosis that would negatively impact the viral lifecycle. Latent KSHV proteins can also activate the DDR by inducing cellular proliferation while LANA plays a role in abrogating cell cycle checkpoints. KSHV also induces DNA damage during lytic replication and several DDR factors associate with replicating viral DNA. 

In both EBV and KSHV, it is problematic to link DNA damage during lytic replication with cellular transformation, since this phase of the viral lifecycle inevitably results in destruction of the host cell. However, several lytic proteins are also expressed briefly following initial viral entry and could conceivably have a detrimental impact on chromosomal stability at an earlier stage of the viral lifecycle [175]. In addition, expression of early lytic proteins during abortive lytic replication of EBV has been linked to the development of lymphomas in mice [176]. Being the most recent human herpesvirus to be identified, there are fewer reports concerning the interaction between KSHV and the DDR compared with other members of the same family. Future studies may focus on identifying a role for DDR activation during lytic replication and the relative contribution of DDR modulation to development of KSHV-related malignancies.

## 9. Human T-cell Leukemia Virus Type 1 (HTLV-1)

Human T-cell leukemia virus (HTLV-1), the only known human retrovirus that can directly transform human cells, is responsible for the rare and aggressive cancer known as adult T-cell-leukemia/lymphoma (ATL) [177]. HTLV-1 is an enveloped virus with a genome composed of two identicalplus-sense single-stranded RNA molecules that are synthesised into a DNA provirus that integrates into the host genome. Like all retroviruses, the HTLV-1 genome contains gag, pol, and ENV structural genes encompassed by two long terminal repeat (LTR) sequences. Bordering the 3′ LTR is the unique pX region that encodes regulatory proteins Tax, Rex, p12, p13, p30, p21, and HTLV-1 basic Zip factor (HBZ) [178].

Like HIV, HTLV-1 predominately targets CD4+ T cells but, while HIV infection can lead to CD4 depletion, HTLV-1-related diseases are characterised by the unregulated proliferation of these lymphocytes. The 40 kDa Tax protein is a transcriptional activator that is essential for viral replication and implicated in HTLV-1-induced cellular transformation [179,180,181]. Tax can stimulate cellular proliferation and survival through modulation of cell cycle checkpoints, stimulation of the NF-kB pathway and activation of the hTERT promoter in quiescent T cells [182]. In addition, Tax can interfere with tumour suppressor function through the inactivation of p53 [183] and proteasomal degradation of Rb [184]. Tax can also form foci, known as Tax Speckled Structures (TSS) [185] or Tax nuclear bodies [186], in the nuclei of infected cells.

### 9.1. Genetic Instability in Tax-Expressing Cells

Multiple groups have reported markers of genomic instability in host cells following expression of Tax, characterised by the prevalence of DNA damage markers, formation of micronuclei, and occurrence of aneuploidy [187,188,189]. Tax was shown to sensitise cells with deleted or mutant p53 to a range of DNA damaging agents including mitomycin C, etoposide and UV light [190]. Expression of Tax can also increase levels of intracellular ROS, DNA damage and the senescence marker SEN1 in both fibroblasts and T cells [191]. All of these markers were reduced following siRNA depletion of Tax or treatment with the ROS scavenger *N*-acetyl-L-cysteine (NAC), indicating that ROS production was the primary source of Tax-induced DNA damage in these cells. Tax has also been shown to impair DNA replication fork progression causing formation of DSBs during S-phase [192]. In the same study, NF-kB activation by Tax also led to DSBs via the production of nitric oxide (NO).

### 9.2. Interference with DNA Repair by HTLV-1 Proteins

Tax expression has been shown to increase the frequency of mutations in the cellular genome [193]. The random nature of these mutations indicates that this viral protein could interfere with the repair of DNA damage that accumulates during normal cellular processes. Tax has since been shown to interfere directly and indirectly with several different repair pathways that include BER, NER, MMR, NHEJ and HR.

The presence of Tax reduces the expression of human polymerase β, an enzyme involved in BER [52]. Using a range of DNA-damaging agents and a plasmid reactivation assay it was shown that base-excision repair of oxidative damage was the primary repair pathway suppressed in Tax-expressing cells [188]. Tax has also been demonstrated to repress NER through transactivation of PCNA, a cofactor for DNA polymerase δ, that plays a central role in DNA replication and repair [54,194]. In cells containing *wt* p53, Tax has a dose-dependent dual effect on NER [195]. Low levels of Tax stimulated NER through increased transcriptional activity of p53 while high levels of Tax increased p53 levels but functionally inactivated the protein leading to inhibition of p53-dependent NER.Alteredexpression of several MMR genes has also been recorded in primary leukemic cells derived from patients with ATL [61]. Expression of MSH2 and PMS1 was decreased in all cases examined and the reduction in the latter was ascribed to increased methylation of the PMS1 promoter.

Following IR, Tax-expressing cells display defects in the formation of DNA repair foci including disruption of the association between MDC1 and γH2AX and reduced association between ATM and chromatin [196]. As a result of impaired ATM activity, Tax-expressing cells progressed more rapidly into S-phase despite the presence of unrepaired DNA damage. A subsequent study demonstrated that MDC1 is bound by Tax and recruited to nuclear foci that also contain DNA-PK and BRCA1 [66]. Formation of these Tax-containing foci led to a reduction in IR-induced foci containing NBS1 suggesting that viral sequestration of DDR factors impedes their involvement in the normal DDR. Tax has also been found to enhance the expression of the cellular phosphatase WIP1 leading to a reduction in levels of γH2AX following UV radiation [197]. Interference with γH2AX accumulation allows Tax expressing cells to bypass the G1/S checkpoint and enter S-phase with unrepaired DNA damage.

Expression of Tax has been shown to directly induce DSBs during S-phase while NF-kB activation by Tax leads to suppression of HR [189]. Tax also represses transcription of the NHEJ protein Ku80 [198,199]. Reduced Ku80 expression increased the number of unprotected DNA breaks as well as the occurrence of micronuclei and nucleoplasmic bridges in Tax-expressing cells. Although the majority of studies investigating genetic instability during HTLV-1 infection have focused on the activitiesof Tax, there is evidence that HTLV-1 RNA binding protein p30 can also interfere with repair of DSBs [200]. Following DNA damage, p30 alters its distribution from the nucleolus to the nucleoplasm and binds to NBS1 and Rad50, which interferes with the formation of the MRN complex. The disruption of the MRN complex by p30 results in impaired HR during S-phase and facilitates a switch to the more error-prone NHEJ.

### 9.3. Interaction between Tax and Checkpoint Kinases

Expression of Tax has been shown to induce ATM-CHK2 signalling resulting in the accumulation of cells in the G2 cell cycle phase [201]. Tax can interact directly with CHK2 and, following DDR activation, they, along with 53BP1, have been observed co-localised in nuclear foci [201]. Another study confirmed the interaction between Tax and CHK2 and demonstrated that Tax can also bind to CHK1 [202]. Through this interaction, Tax was shown to inhibit the kinase activity of CHK1 preventing the degradation of Cdc25A and impairing G2 arrest following IR. A subsequent publication attempted to address these contradictory findings by demonstrating that Tax can interact with the kinase domain of CHK2 stimulating oligomerization, autophosphorylation, and stabilisation of the protein [203]. Following IR, Tax sequesters the phosphorylated form of CHK2 within chromatin and impairs its role in the DDR following IR treatment. This study concluded that following Tax expression, CHK2 maintains the ability to orchestrate cell cycle arrest in G2 but is impaired in its role in the DDR following IR. DNA-PK has also been observed associated with nuclear foci containing Tax and CHK2 [204]. Tax can mediate an interaction between DNA-PK and CHK2 leading to increased DNA-PK activity, which impairs the cellular response to IR. 

### 9.4. Summary

Multiple groups have shown that expression of HTLV-1 Tax can cause DNA damage and that this can occur via several mechanisms including the generation of free radicals and interference with DNA replication. There is evidence that at least five key DNA repair pathways are compromised during HTLV infection and that the proper formation of repair complexes is disrupted. In addition, checkpoint kinases are targeted and the virus modulates cell cycle progression to maximise viral replication efficiency.

Following infection, HTLV-1 can enter a long period of asymptomatic latency with only 3%–4% of infected individuals eventually developing ATL [205]. This suggests that cancer development and progression following HTLV-1 infection requires the accumulation of multiple genetic changes [206]. By introducing DNA damage and interfering with repair pathways and cell cycle checkpoints, HTLV-1 can induce cellular proliferation in the presence of genetic aberrations that could ultimately result in cellular transformation. 

## 10. Hepatitis B virus (HBV)

Hepatitis B virus (HBV) is a small enveloped DNA virus with a 3.2 kb genome belonging to the *Hepadnaviridae* family. HBV targets hepatocytes and, while transient infection can cause acute hepatitis, chronic infection is a major risk factor for the development of hepatocellular carcinoma (HCC). The circular HBV genome is partially double stranded and contains four ORFs named S, C, P, and X. The S gene, also known as HBsAg, is divided into Pre-S1, Pre-S2, and S domains and encodes three envelope proteins often referred to as large (L), middle (M), and small (S). The three remaining ORFs encode a DNA polymerase (Pol), a capsid protein (core) and the 154 amino acid X protein (HBx) [207].

HBV has a relatively complex lifecycle that involves reverse transcription. Following entry to the cell, the HBV relaxed circular DNA (rcDNA) is transported to the nucleus where it is converted into covalently closed circular DNA (cccDNA). The cccDNA provides a template for production of viral RNA that is converted by reverse transcriptase to HBV rcDNA in the cytoplasm. Although not an essential step in the viral lifecycle, the HBV genome is often found integrated into the host genome and the process has been proposed to play a role in hepatocarcinogenesis [208].

The multifunctional HBx is involved in viral replication and is implicated in the development of HCC due to its ability to interfere with multiple cellular processes [209]. In the cytoplasm HBx can interact with mitochondria and activate mitogenic signalling cascades [209]. In the nucleus, HBx can influence gene expression through interaction with cellular transcription machinery, although it does not bind DNA directly [210]. HBx can also interact with p53 and impair its transcriptional activation of target genes, thus interfering with its role in apoptotic and DNA repair pathways [211,212,213,214,215].

### 10.1. Introduction of Oxidative DNA Damage by HBV

Chronic HBV infection can result in increased oxidative stress that has been linked to development of HBV-associated liver disease [216]. Using transgenic mice that expressed the HBV large envelope protein in hepatocytes, it was shown that development of chronic liver disease is associated with an increase in oxidative DNA damage [217]. Mutations in the Pre-S region of the HBV surface antigen (HBsAg) are associated with increased ROS production via endoplasmic reticulum (ER) stress [218,219]. Expression of pre-S mutanT antigens in hepatoma cell lines was shown to cause increased oxidative DNA damage and increase expression of the DNA repair gene ogg1 [218]. However, a subsequent study failed to find a correlation between HBV pre-S mutations and increased oxidative DNA damage in patients with HCC [220]. 

It has also been demonstrated that DNA damage caused by oxidative stress can promote HBV integration and this has been linked to the development of HCC [221]. Inhibition of PARP-1, involved in SSB repair, also increased the occurrence of HPV genome integration indicating that functional DNA repair pathways, as well as limiting the impact of oxidative DNA damage, may protect against tumour development by restricting viral integration frequency.

### 10.2. Interference with DNA Repair by HBV

As well as promoting oxidative damage, HBV proteins have been shown to interfere with DNA repair pathways including those associated with processing of ROS-induced DNA lesions. HBx is known to bind to DNA binding protein 1 (DDB1) and the interaction has been shown to be important for HBV replication [56,57,222,223]. As well as being a subunit of an E3 ubiquitin ligase complex, DDB1 also plays a role in both GG-NER and TC-NER by forming complexes with DDB2 and Cockayne syndrome group A protein, respectively [224]. Using a panel of HBx mutants it was shown that, while HBx can impede repair of UV-damage, the interaction between HBx-DDB1 was not absolutely required for this effect [225]. Although it has not been directly linked to impaired NER, the interaction between HBx and DDB1 interferes with normal S-phase progression and increases the incidence of lagging chromosomes during mitosis that can subsequently cause multi-nucleation [226]. 

HBx, through its interaction with the Sp1 transcription factor, can also reduce expression of the XPD and XPB subunits of the TFIIH transcription factor involved in NER [227]. In addition, HBx has been shown to interact directly with XPD and XPB and sensitise cells to UV-induced DNA damage [58,59]. The interaction between HBx and p53 has been linked to defects in NER and expression of HBx can inhibit p53-dependent GG-NER in primary mouse hepatocytes [228]. Following UV exposure, levels of HBx and p53 were shown to increase in a hepatocellular carcinoma cell line and both proteins co-localized in the nucleus [229]. The HBx-expressing cells also displayed increased G2/M arrest and apoptosis, as well as a reduced capacity to repair DNA damage compared with controls. HBx expression also impedes the other NER sub-pathway, TC-NER, in both *wt* and p53-null cell lines [215].

Although the majority of studies regarding HBV and DNA repair have focused on the NER pathway, there is also evidence that HBx can interfere with BER. The structure of HBx has been shown to be similar to the human thymine DNA glycosylase (TDG) involved in initiating BER [230]. While the presence of TDG does not affect viral replication, HBx expression strongly supresses BER of G/T mismatches normally initiated by TDG. A more recent study assessed the individual capacity of HBsAg, core protein and HBx to induce DNA damage and interfere with DNA repair [231]. Accumulation of HBsAg was shown to enhance degradation of PML, which led to delayed repair of DSBs and increased resistance to apoptosis following IR. The results also showed that while HBx can induce DNA damage and apoptosis, it did not impede the repair of IR-induced lesions.

### 10.3. Activation of the ATR Pathway by HBx

HBx expression has been demonstrated to activate the ATR pathway [232,233,234]. HBV infection can activate the ATR-CHK1 pathway with minimal effect on ATM-CHK2 [233]. Expression of HBx alone has been shown to induce DNA re-replication and polyploidy through increased expression of Cdc6 and Cdt1 and down-regulation of geminin [234]. DNA re-replication resulted in increased DNA damage and the activation of ATR leading to phosphorylation of Rad17 and H2AX but not CHK1. It was also demonstrated that HBx-expressing cells are able to progress to mitosis despite the presence of DNA damage [235]. Activation of Polo-like kinase 1 (Plk1) in the G2 phase of the cell cycle by HBx reduced levels of Claspin, leading to CHK1 inactivation. This led to attenuation of the G2/M cell cycle checkpoint and subsequently suppressed DNA repair and p53-mediated apoptosis. Treatment of HBV infected cells with theophylline, a compound that inhibits ATR and ATM signalling, significantly reduces HBV replication suggesting a role for DDR signalling in the HBV lifecycle [236].

### 10.4. Summary

It is clear that HBV infection can increase oxidative stress leading to elevated levels of DNA damage in infected hepatocytes. HBV also appears to interfere with several DNA repair pathways, primarily through the actions of HBx, potentially exacerbating the increase in DNA damage. In addition, HBx can activate the ATR pathway, interfere with checkpoint activation and promote cell cycle progression in the presence of DNA damage that could ultimately result in genetic aberrations.

Despite BER being the primary pathway for repair of oxidative DNA damage, the majority of reports concerning HBV and DNA repair have focused on NER. Although NER can play a part in repair of oxidative damage, it is still unclear whether modulation of this pathway has a substantial role in tumour development following viral infection. As with HPV, DNA damage and abrogated repair can increase the likelihood of HBV integration, which has been linked to deleterious genetic alterations and altered expression of cellular genes. The fact that 80%–90% of HBV-related HCCs contain integrated HBV sequences highlights the link between viral integration and tumourigenesis [237]. 

## 11. Hepatitis C virus (HCV)

Belonging to the *Flaviviridae* family, the Hepatitis C virus (HCV) chronically infects over 170 million people worldwide and is responsible for around a third of cases of HCC [238]. While primarily a hepatotrophic virus, HCV can also replicate in lymphocytes and has been linked to several lymphoproliferative disorders including B-cell non-Hodgkin’s lymphoma (NHL) [239].

HCV has a positive-sense single-stranded RNA genome of 9.6 kb that contains 5′ and 3′ untranslated regions (UTRs) that border a single ORF. An inteRNAl ribosome entry site (IRES) in the 5' UTR initiates translation of the HCV genome. The HCV ORF encodes a viral polyprotein of approximately 3010 amino acids that is subsequently cleaved by host proteases to produce a core protein, the E1 and E2 glycoproteins and the p7 ion channel protein. Additional cleavage by viral proteases produces six non-structural proteins NS2, NS3, NS4A, NS4B, NS5A, and NS5B. 

Like all positive-strand RNA viruses, HCV reorganises intracellular membranes, such as those associated with mitochondria or endoplasmic reticulum, to form replication structures in the cytoplasm known as “membranous webs” [240,241]. Remodelling of endoplasmic reticulum has been observed following expression of NS4B suggesting that this protein is primarily responsible for the formation of HCV replication complexes [240].

### 11.1. Introduction of Oxidative DNA Damage by HCV

As with HBV, infection with HCV can lead to elevated levels of oxidative stress that subsequently increase the occurrence of DNA damage. Expression of HCV core and NS3 proteins have been shown to stimulate the inducible NO synthase (iNOS) gene leading to increased production of nitric oxide (NO) and formation of DSBs in cellular DNA [242]. Expression of E1 during the study also caused a moderate increase in DSB formation but by a mechanism independent of NO production. It was subsequently demonstrated that HCV infection leads to a reduction in mitochondrial membrane potential and an increase in ROS levels [243]. Individual expression of core, E1, and NS3 confirmed that each protein is able to induce ROS that subsequently leads to an increase in DSB formation. 

HCV infection can also lead to overexpression of 3β-hydroxysterol Δ24-reductase (DHCR24) which has been linked to attenuation of the p53-mediated response to oxidative stress [244]. Activation of Akt by NS5A can increase c-Myc transcription through stabilisation of the transcription factor β-catenin [245]. This increased expression of c-Myc enhances ROS production and results in increased DNA damage and cell cycle arrest.

### 11.2. Interference with DNA Repair by HCV

Several studies have demonstrated that interference with DNA repair pathways by HCV could contribute to the development of HCC. Reduced expression of several genes involved in the MMR pathway including MSH2, MLH1, GTBP and PMS2, has been observed in HCV-positive tissues from patients with HCC [246]. Another study demonstrated that ROS levels were elevated by 30–60 fold in HCV HCC cells compared to uninfected controls and that expression of the DNA glycosylase NEIL1, involved in the initial steps of BER, was suppressed [247]. Treatment with the antioxidant NAC or antiviral interferon (IFN) led to a partial restoration in NEIL1 expression indicating that virally-induced ROS could potentially impair BER. Down-regulation of Gadd45β, that plays a role in NER, was observed in HCV-infected cell lines and tissues, as well as transgenic mice expressing the entire HCV ORF [248]. Hypermethylation of the Gadd45β promoter in the presence of HCV was found to be responsible for the defect and led to impaired cell cycle arrest and reduced DNA excision repair. It has also been shown that expression of the HCV core protein in hepatocellular carcinoma cells leads to an impaired ability to repair UV-induced DNA damage [249].

As well as attenuating excision repair pathways, HCV can also interfere with DSB repair. Infection of lymphocytes with HCV was found to cause chromosomal aberrations, which are at least partially attributed to elevated NO and ROS levels [250]. In the same study, expression of Core and NS3 HCV proteins sensitised cells to DNA damaging agents by inhibiting NHEJ. In addition, HCV core protein was shown to interact with NBS1 and prevent formation of the MRN complex, which impairs ATM-mediated repair of DSBs. 

### 11.3. Interaction between HCV Proteins and the ATM Pathway

In addition to inducing DNA damage, HCV can also modulate the DDR through interaction with the ATM-CHK2 pathway. NS3/4A can interact directly with ATM while NS5B interacts with both ATM and CHK2 [251]. In the same study, knockdown of ATM and CHK2 reduced replication of HCV RNA suggesting a functional ATM pathway is required for efficient viral replication. The interaction between NS3/4A and ATM was confirmed in a separate study and shown to sensitise cells to IR via retention of ATM in the cytoplasm that subsequently impairs phosphorylation of H2AX following DNA damage [252]. In addition, expression of the HCV non-structural transmembrane protein NS2 can enhance cellular proliferation and activate the ATM-CHK2 pathway [253]. Following DDR activation, p53 was retained in the cytoplasm in NS2-expressing cells resulting in impaired downstream signalling through p21 activation.

### 11.4. Summary

As with HBV, infection with HCV can lead to elevated intracellular oxidative stress that increases the occurrence of DNA damage. HCV proteins can also interact with several DDR factors resulting in a reduced capacity to repair DNA lesions. Expression of HCV proteins activates the ATM pathway although the virus appears to deregulate downstream signalling while using key components for viral replication. There is also evidence that HCV exploits the DDR to modulate cell cycle progression [254,255]. NS5B was shown to interact with cyclin-dependent kinase 2-interacting protein (CINP), involvedinthe ATR-CHK1 pathway, preventing its localisation to the nucleus and ultimately causing a delay in S-phase progression [255]. 

Despite having an RNA genome and replicating in the cytoplasm, HCV can still impact significantly on host DDR pathways. While the HCV lifecycle does not include a nuclear stage, truncated or mutated versions of several HCV proteins have been observed in the nucleus where they can interact with cellular proteins including those associated with the DDR [250,256,257]. There is some doubt, however, about the nuclear localisation of these proteins during realistic models of infection [258]. To this end, the development of superior murine models of HCV pathogenesis will be invaluable in assessing the relative contribution of DDR deregulation and other factors to the progression of HCC.

## 12. Conclusions

It is clear from the literature summarised here that the interaction between human tumour viruses and the DDR can contribute to an increase in genomic instability during viral infection. As is apparent in high-risk HPV types, the accumulation of deleterious mutations in the presence of viral oncoproteins that promote cellular survival and proliferation will inevitably increase the likelihood of cellular transformation. While the DDR can represent an antiviral defence and barrier to tumourigenesis, chronic activation also creates selection pressure for the deactivation of key components. In all the viruses covered here, DDR activation is an inevitable consequence of viral infection and can be the direct result of the recognition of foreign DNA or expression of viral proteins, or can occur more indirectly through elevated oxidative stress or aberrant cellular proliferation. Since cell cycle arrest and apoptosis are consequences of DDR activation, viruses typically inhibit downstream signalling that would have negative consequences for viral propagation. It is also notable that, in HBV and HPV, DNA damage inflicted during infection can increase the chances of viral integration. This, in turn, can contribute to malignant transformation through host genomic alterations and elevated expression of viral oncogenes [259].

While there have been many excellent studies in this field, in certain cases, experimental limitations have led to some contradictory findings that have impeded progress. The use of different cell culture models and non-specific kinase inhibitors has resulted in inconsistent conclusions regarding the role of DDR activation during replication of viruses such as EBV. A more detailed mechanistic understanding of the precise roles of DDR proteins during synthesis of viral genomes will help confirm their importance during the viral lifecycle. While the ectopic expression of viral genes is a typical way of assessing their contribution to DDR deregulation, it may be misleading in cell types that are not naturalviraltargets *in vivo*. In the case of EBV, for example, the BZLF1 lytic protein has contrasting effects on cell cycle progression when expressed in different cell lines [260]. It is also apparent that viral proteins may have dual effects on the DDR depending on levels of expression; an example mentioned above is the effect of HTLV-1 Tax on p53-dependent NER [195]. In these cases, results must be considered in the context of gene expression levels during natural viral infection. Furthermore, possible contributory effects of other viral proteins should be borne in mind.

Reports demonstrating the involvement of DDR factors in viral replication have led to suggestions that these proteins could be targeted as a novel therapeutic strategy [251]. This is particularly appealing in the case of viruses with RNA genomes where higher rates of mutation have limited efforts to target the virus directly [261]. In addition, it has also been proposed that inactivation of key DNA repair pathways by viruses could open the door for the use of synthetic lethality to specifically target tumours containing viral genomes [262]. While these are attractive possibilities, the DDR is a rapidly evolving area of research with new components and novel regulatory mechanisms being identified frequently. Virologists must keep abreast of the most recent findings to gain a more complete understanding of how viruses modulate these pathways. While it remains to be seen whether the study of viruses and the DDR will yield effective therapeutic interventions, at the very least it has shed new light on how these pathogens modulate host cell functions and uncovered new facets of DDR regulation.
