# Correction: Hollingworth, R.; Grand, R.J. Modulation of DNA Damage and Repair Pathways by Human Tumour Viruses. *Viruses* 2015, *7*, 2542-2591

**DOI:** 10.3390/v7062767

**Published:** 2015-06-19

**Authors:** Robert Hollingworth, Roger J. Grand

**Affiliations:** School of Cancer Sciences, College of Medicine and Dentistry, University of Birmingham, Birmingham B15 2TT, UK; E-Mail: rxh291@student.bham.ac.uk

We have noted a number of errors in the references of this manuscript. Please find below the list of corrected references:

3. Turnell, A.S.; Grand, R.J. DNA viruses and the cellular DNA-damage response. *J. Gen. Virol.* **2012**, *93*, 2076–2097.
4. Marriott, S.J.; Semmes, O.J. Impact of HTLV-I tax on cell cycle progression and the cellular DNA damage repair response. *Oncogene* **2005**, *24*, 5986–5995.
5. Smith, J.A.; Daniel, R. Following the path of the virus: The exploitation of host DNA repair mechanisms by retroviruses. *ACS Chem. Biol.* **2006**, *1*, 217–226.
6. Higgs, M.R.; Chouteau, P.; Lerat, H. 'Liver let die': Oxidative DNA damage and hepatotropic viruses. *J. Gen. Virol.* **2014**, *95*, 991–1004.
9. Sinclair, A.; Yarranton, S.; Schelcher, C. DNA-damage response pathways triggered by viral replication. *Expert Rev. Mol. Med.* **2006**, *8*, 1–11.
12. Parkin, D.M. The global health burden of infection-associated cancers in the year 2002. *Int. J. Cancer* **2006**, *118*, 3030–3044.
33. Davy, C.; Doorbar, J. G2/m cell cycle arrest in the life cycle of viruses. *Virology* **2007**, *368*, 219–226.
119. Duensing, S.; Munger, K. The human papillomavirus type 16 e6 and e7 oncoproteins independently induce numerical and structural chromosome instability. *Cancer Res.* **2002**, *62*, 7075–7082.
135. Boichuk, S.; Hu, L.; Hein, J.; Gjoerup, O.V. Multiple DNA damage signaling and repair pathways deregulated by simian virus 40 large T antigen. *J. Virol.* **2010**, *84*, 8007–8020.
138. Maeda, E.; Akahane, M.; Kiryu, S.; Kato, N.; Yoshikawa, T.; Hayashi, N.; Aoki, S.; Minami, M.; Uozaki, H.; Fukayama, M.; *et al.* Spectrum of epstein-barr virus-related diseases: A pictorial review. *Jpn. J. Radiol.* **2009**, *27*, 4–19.
163. Ballestas, M.E.; Chatis, P.A.; Kaye, K.M. Efficient persistence of extrachromosomal kshv DNA mediated by latency-associated nuclear antigen. *Science* **1999**, *284*, 641–644.
207. Miller, R.H.; Kaneko, S.; Chung, C.T.; Girones, R.; Purcell, R.H. Compact organization of the hepatitis b virus genome. *Hepatology* **1989**, *9*, 322–327.
208. Edman, J.C.; Gray, P.; Valenzuela, P.; Rall, L.B.; Rutter, W.J. Integration of hepatitis b virus sequences and their expression in a human hepatoma cell. *Nature* **1980**, *286*, 535–538.
209. Wei, Y.; Neuveut, C.; Tiollais, P.; Buendia, M.A. Molecular biology of the hepatitis B virus and role of the x gene. *Pathol. Biol.* **2010**, *58*, 267–272.
214. Mathonnet, G.; Lachance, S.; Alaoui-Jamali, M.; Drobetsky, E.A. Expression of hepatitis b virus x oncoprotein inhibits transcription-coupled nucleotide excision repair in human cells. *Mutat. Res.* **2004**, *554*, 305–318.
215. Knoll, S.; Furst, K.; Thomas, S.; Villanueva Baselga, S.; Stoll, A.; Schaefer, S.; Putzer, B.M. Dissection of cell context-dependent interactions between hbx and p53 family members in regulation of apoptosis: A role for hbv-induced hcc. *Cell Cycle* **2011**, *10*, 3554–3565.
216. Bolukbas, C.; Bolukbas, F.F.; Horoz, M.; Aslan, M.; Celik, H.; Erel, O. Increased oxidative stress associated with the severity of the liver disease in various forms of hepatitis b virus infection. *BMC Infect. Dis.* **2005**, *5*, e95.
219. Wang, H.C.; Huang, W.; Lai, M.D.; Su, I.J. Hepatitis b virus pre-s mutants, endoplasmic reticulum stress and hepatocarcinogenesis. *Cancer Sci.* **2006**, *97*, 683–688.
220. Gwak, G.Y.; Lee, D.H.; Moon, T.G.; Choi, M.S.; Lee, J.H.; Koh, K.C.; Paik, S.W.; Park, C.K.; Joh, J.W.; Yoo, B.C. The correlation of hepatitis b virus pre-s mutation with cellular oxidative DNA damage in hepatocellular carcinoma. *Hepatogastroenterology* **2008**, *55*, 2028–2032.
223. Hodgson, A.J.; Hyser, J.M.; Keasler, V.V.; Cang, Y.; Slagle, B.L. Hepatitis b virus regulatory hbx protein binding to ddb1 is required but is not sufficient for maximal hbv replication. *Virology* **2012**, *426*, 73–82.
224. Iovine, B.; Iannella, M.L.; Bevilacqua, M.A. Damage-specific DNA binding protein 1 (ddb1): A protein with a wide range of functions. *Int. J. Biochem. Cell Biol.* **2011**, *43*, 1664–1667.
237. Sung, W.K.; Zheng, H.; Li, S.; Chen, R.; Liu, X.; Li, Y.; Lee, N.P.; Lee, W.H.; Ariyaratne, P.N.; Tennakoon, C.; *et al.* Genome-wide survey of recurrent hbv integration in hepatocellular carcinoma. *Nat. Genet.* **2012**, *44*, 765–769.
238. Shepard, C.W.; Finelli, L.; Alter, M.J. Global epidemiology of hepatitis c virus infection. *Lancet Infect. Dis.* **2005**, *5*, 558–567.
239. Zignego, A.L.; Macchia, D.; Monti, M.; Thiers, V.; Mazzetti, M.; Foschi, M.; Maggi, E.; Romagnani, S.; Gentilini, P.; Brechot, C. Infection of peripheral mononuclear blood cells by hepatitis c virus. *J. Hepatol.* **1992**, *15*, 382–386.
240. Egger, D.; Wolk, B.; Gosert, R.; Bianchi, L.; Blum, H.E.; Moradpour, D.; Bienz, K. Expression of hepatitis c virus proteins induces distinct membrane alterations including a candidate viral replication complex. *J. Virol.* **2002**, *76*, 5974–5984.
241. Gosert, R.; Egger, D.; Lohmann, V.; Bartenschlager, R.; Blum, H.E.; Bienz, K.; Moradpour, D. Identification of the hepatitis c virus rna replication complex in huh-7 cells harboring subgenomic replicons. *J. Virol.* **2003**, *77*, 5487–5492.
257. Maqbool, M.A.; Imache, M.R.; Higgs, M.R.; Carmouse, S.; Pawlotsky, J.M.; Lerat, H. Regulation of hepatitis c virus replication by nuclear translocation of nonstructural 5a protein and transcriptional activation of host genes. *J. Virol.* **2013**, *87*, 5523–5539.
